# Impact of anemia on clinical outcomes of patients with atrial fibrillation: The COOL‐AF registry

**DOI:** 10.1002/clc.23559

**Published:** 2021-02-04

**Authors:** Rungroj Krittayaphong, Satchana Pumprueg, Tomon Thongsri, Weerapan Wiwatworapan, Thaworn Choochunklin, Pontawee Kaewkumdee, Ahthit Yindeengam, for the COOL‐AF Investigators

**Affiliations:** ^1^ Division of Cardiology, Department of Medicine, Faculty of Medicine Siriraj Hospital Mahidol University Bangkok Thailand; ^2^ Department of Cardiology Buddhachinaraj Hospital Phitsanulok Thailand; ^3^ Department of Cardiology Maharat Nakorn Ratchasima Hospital Nakorn Ratchasima Thailand; ^4^ Deparment of Cardiology Surin Hospital Surin Thailand

**Keywords:** anemia, atrial fibrillation, bleeding, clinical outcomes, ischemic stroke, Thailand

## Abstract

**Background:**

To determine whether anemia is an independent risk factor for ischemic stroke and major bleeding in patients with non‐valvular atrial fibrillation (NVAF).

**Hypothesis:**

Anemia in patients with NVAF increase risk of clinical complications related to atrial fibrillation.

**Methods:**

We conducted a prospective multicenter registry of patients with NVAF in Thailand. Demographic data, medical history, comorbid conditions, laboratory data, and medications were collected and recorded, and patients were followed‐up every 6 months. The outcome measurements were ischemic stroke or transient ischemic attack (TIA), major bleeding, heart failure (HF), and death. All events were adjudicated by the study team. We analyzed whether anemia is a risk factor for clinical outcomes with and without adjusting for confounders.

**Results:**

There were a total of 1562 patients. The average age of subjects was 68.3 ± 11.5 years, and 57.7% were male. The mean hemoglobin level was 13.2 ± 1.8 g/dL. Anemia was demonstrated in 518 (33.16%) patients. The average follow‐up duration was 25.8 ± 10.5 months. The rate of ischemic stroke/TIA, major bleeding, HF, and death was 2.9%, 4.9%, 1.8%, 8.6%, and 9.2%, respectively. Anemia significantly increased the risk of these outcomes with a hazard ratio of 2.2, 3.2, 2.9, 1.9, and 2.8, respectively. Oral anticoagulants (OAC) was prescribed in 74.8%; warfarin accounts for 89.9% of OAC. After adjusting for potential confounders, anemia remained a significant predictor of major bleeding, heart failure, and death, but not for ischemic stroke/TIA.

**Conclusion:**

Anemia was found to be an independent risk factor for major bleeding, heart failure, and death in patients with NVAF.

## 1 INTRODUCTION

Non‐valvular atrial fibrillation (NVAF) is the leading cause of ischemic stroke.^1^ Oral anticoagulants (OACs) are the standard treatment for stroke prevention in patients with risk factors, including the elderly, and those with diabetes or hypertension.^2^, ^3^, ^4^ Anemia is detected in approximately 21% of subjects older than 65 years, and in 31% of those older than 80 years.^5^ Among patients with NVAF, anemia was detected in 13–34%.^6^, ^7^ OAC treatment in patients with anemia may increase the risk of bleeding since some patients may have a hidden site of bleeding.^6^, ^8^ Moreover, some causes of anemia may increase the risk of bleeding, such as concomitant thrombocytopenia or platelet dysfunction.

Compared to the decision whether or not to use OAC in non‐anemic NVAF, the decision to prescribe OAC in NVAF patients with anemia will depend on the cause and severity of anemia.^7^ Moreover, when using OAC, anemia may affect the choice of OAC.^6^, ^8^, ^9^ When warfarin is used, physicians tend to maintain a low international normalized ratio (INR) target, which often leads to a suboptimal time in therapeutic range (TTR).^7^

The aim of this study was to determine whether anemia is an independent risk for ischemic stroke/transient ischemic attack (TIA) and major bleeding in patients with NVAF.

## 2 METHODS

### 2.1 Study population

Patients aged 18 years or older who were diagnosed with NVAF were eligible for enrollment. The exclusion criteria were: (1) ischemic stroke within 3 months; (2) hematologic diseases that increase risk of bleeding, such as thrombocytopenia or myeloproliferative disorders; (3) rheumatic valvular disease; (4) mechanical heart valve or valve repair; (5) atrial fibrillation from transient reversible cause; (6) life expectancy less than 3 years; (7) inability to attend scheduled follow‐up; (8) refusal to participate; (9) current participation in a clinical trial; and, (10) no baseline hemoglobin data. The study protocol was mentioned earlier.^10^

This study was approved by the Institutional Review Board (IRB) of each participating hospital, and the IRB of the Thailand Ministry of Public Health (MOPH). All patients signed written informed consent prior to participation. The subjects were enrolled from 27 hospitals in Thailand during June 2014 to October 2017.

### 2.2 Study protocol

After informed consent was obtained, baseline data were recorded onto a case record form (CRF) using data from both the medical record and patient interview. All data in the CRF were then uploaded into the web system. All CRFs were sent to the data management site for centralized data management, including double entry, and validation of data. All questions concerning the correctness of data were sent to the site staff for confirmation. Follow‐up data were recorded in a similar manner every 6 months until 3 years. Site monitoring was performed at every site to ensure site staff compliance with the study protocol.

### 2.3 Data collection

The following data were collected at baseline: (1) demographic data; (2) medical history and comorbid conditions (e.g., diabetes, hypertension, smoking status, history of coronary artery disease [CAD], history of heart failure); (3) history of ischemic stroke/TIA and history of bleeding; (4) type and duration of atrial fibrillation; (5) components of CHA_2_DS_2_‐VASc and HAS‐BLED scores; (6) vital signs; (7) medications including, OAC and antiplatelet; (8) twelve‐lead electrocardiogram (ECG); and, (9) laboratory data within 6 months, including hemoglobin and INR. Anemia was defined by World Heart Organization (WHO) criteria as a hemoglobin level < 13 g/dl for male, and < 12 g/dl for female.^11^

Clinical outcomes, components of CHA_2_DS_2_‐VASc and HAS‐BLED scoring, medications, and laboratory data were recorded at each 6‐month follow‐up visit. Patients needed at least 6 months of follow‐up data to be included in the analysis.

### 2.4 Outcome measurement

The main outcome measurements were ischemic stroke/TIA, major bleeding, heart failure, and death. Investigators were instructed to upload medical documentation and reports that supported any of these primary outcome events into the registry's web‐based system. All supporting documents were reviewed by the adjudication committee to confirm the reported events. Ischemic stroke was defined as a sudden onset of neurological deficit lasting more than 24 h. TIA was defined in a similar manner, but the deficit lasted less than 24 hours. Major bleeding was defined according to International Society of Thrombosis and Hemostasis (ISTH) criteria.^12^ Major bleeding included fatal bleeding, bleeding in a critical area or organ, and bleeding that requires a transfusion of at least 2 units of red cells and/or a decrease in hemoglobin level of at least 20 g/dl. Heart failure was defined by the presence of signs and/or symptoms of heart failure and lung congestion, and objective findings of left ventricular systolic dysfunction or structural heart disease.

### 2.5 Statistical analysis

Data are described as mean ± SD for continuous data, and as number and percentage for categorical data. Continuous data were compared by Student's *t* test for unpaired data, whereas categorical data were compared by chi‐square test or Fisher's exact test. Cox proportional hazards model was used to test the effect of anemia on clinical outcomes, and the results are shown as hazard ratio (HR) and 95% confidence interval (CI). The effects were adjusted for potential confounders using multivariable analysis with Cox proportional model with the adjustment for age, gender, comorbid conditions (history of ischemic stroke, CAD, HF, hypercholesterolemia, hypertension, cardiac implantable electronic devices, smoking status, history of major bleeding, and renal replacement therapy), and OAC and antiplatelet. Sensitivity analysis was performed by categorizing anemia into 3 groups (no anemia, mild anemia, moderate to severe anemia), and by using anemia as continuous data and analyze the effect of anemia on clinical outcomes. Mild anemia was defined as a hemoglobin level < 13 g/dl for male, and < 12 g/dl for female but ≥11 g/dl. Moderate to severe anemia was defined as a hemoglobin level < 11 g/dl.^11^ Sensitivity analysis was performed in many aspects: (1) by running analysis using hemoglobin as a continuous data instead of using anemia groups; (2) by using average of hemoglobin from every visit to classify anemia; (3) by using last hemoglobin before the clinical events to classified anemia status (anemia‐ updated); and (4) by the adjustment of time varying covariates. Restricted cubic splines graph was used to demonstrate the effect of anemia as continuous data on each clinical outcome. Generalized Estimating Equation (GEE) with exchangeable correlation structure was used to analyze the effect of anemia on clinical outcomes with the adjustment of time varying covariates. A *p*‐value less than .05 was considered statistically significant. The data were analyzed by SPSS Statistics version 23 (SPSS, Inc., Chicago, IL, USA) and R version 3.6.3.

## 3 RESULTS

A total of 1562 patients were enrolled. The mean age was 68.3 ± 11.5 years, and 661 (42.3%) were female. The average hemoglobin level was 13.2 ± 1.8 g/dl. Anemia was demonstrated in 518 (33.16%) patients. Table 1 shows baseline characteristics of the study population compared between those with and without anemia. Patients with anemia were older, were more likely to have permanent atrial fibrillation, diabetes, hypertension, history of CAD, heart failure, renal replacement therapy, history of bleeding, higher CHA_2_DS_2_‐VASc and HAS‐BLED scores, and more use of antiplatelet.

**TABLE 1:** Baseline characteristics of all non‐valvular atrial fibrillation patients, and compared between those with and without anemia

Characteristics	All (*N* = 1562)	Anemia (*n* = 518)	No anemia (*n* = 1044)	*p*‐value
Age (years)	68.3 ± 11.5	72.0 ± 11.0	66.6 ± 11.3	***<.001***
Female gender	661 (42.3%)	213 (41.1%)	448 (42.9%)	.500
Time after diagnosis of AF (years)	3.1 ± 4.5	3.1 ± 4.2	3.1 ± 4.6	.874
Atrial fibrillation				***.010***
Paroxysmal	560 (35.9%)	180 (34.7%)	380 (36.4%)	
Persistent	347 (22.2%)	96 (18.5%)	251 (24.0%)	
Permanent	655 (41.9%)	242 (46.7%)	413 (39.6%)	
Symptomatic atrial fibrillation	1207 (77.3%)	387 (74.7%)	820 (78.5%)	.089
History of heart failure	424 (27.1%)	175 (33.8%)	249 (23.9%)	***<.001***
History of coronary artery disease	280 (17.9%)	124 (23.9%)	156 (14.9%)	***<.001***
Cardiac implantable electronic device	183 (11.7%)	64 (12.4%)	119 (11.4%)	.580
History of TIA/ischemic stroke	302 (19.3%)	107 (20.7%)	195 (18.7%)	.351
Hypertension	1106 (70.8%)	388 (74.9%)	718 (68.8%)	***.012***
Diabetes mellitus	393 (25.2%)	161 (31.1%)	232 (22.2%)	***<.001***
Smoking	348 (22.3%)	115 (22.2%)	233 (22.3%)	.958
Dyslipidemia	904 (57.9%)	291 (56.2%)	613 (58.7%)	.339
Renal replacement therapy	36 (2.3%)	23 (4.4%)	13 (1.2%)	***<.001***
Dementia	18 (1.2%)	7 (1.4%)	11 (1.1%)	.604
History of bleeding	162 (10.4%)	68 (13.1%)	94 (9.0%)	***.012***
CHA_2_DS_2_‐VASc score				***<.001***
0	93 (6.0%)	9 (1.7%)	84 (8.0%)	
1	168 (10.8%)	28 (5.4%)	140 (13.4%)	
≥2	1301 (83.3%)	481 (92.9%)	820 (78.5%)	
HAS‐BLED score				***<.001***
0	197 (12.6%)	30 (5.8%)	167 (16.0%)	
1‐2	1049 (67.2%)	349 (67.4%)	700 (67.0%)	
≥3	316 (20.2%)	139 (26.8%)	177 (17.0%)	
Antiplatelet	439 (28.1%)	179 (34.6)	260 (24.9%)	***<.001***
Anticoagulant	1169 (74.8%)	385 (74.3%)	784 (75.1%)	.741
Warfarin	1039 (66.5%)	351 (67.8%)	688 (65.9%)	.463
NOACs	130 (8.3%)	34 (6.6%)	96 (9.2%)	.076

*Note:* Data presented as mean ± standard deviation or number and percentage. A *p*‐value<.05 indicates statistical significance (bold and italic).

Abbreviations: NOACs, non‐vitamin K antagonist oral anticoagulants; TIA, transient ischemic attack.

### 3.1 Anemia and outcomes

**FIGURE 1:**
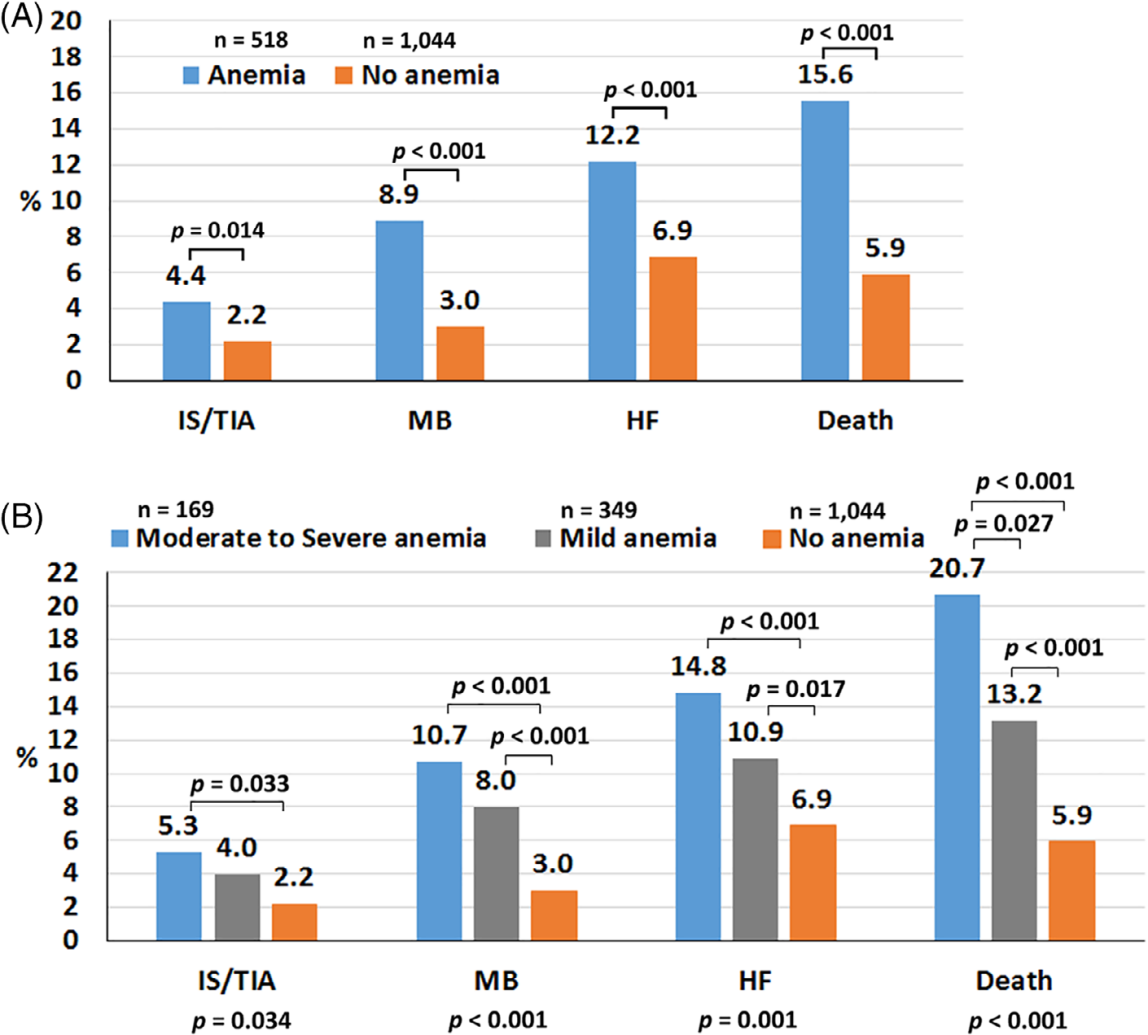
Bar graph showing the rate of ischemic stroke (IS)/transient ischemic attack (TIA), major bleeding (MB), heart failure (HF), and death according to anemia status. Anemia was compared between anemia and no anemia in panel A (upper panel), and compared among moderate or severe anemia, mild anemia, and no anemia in panel B (lower panel). A *p*‐value <.05 indicates statistical significance

The average follow‐up duration was 25.8 ± 10.5 months. Rate of ischemic stroke/TIA, major bleeding, heart failure, and death was 1.39%, 2.33%, 0.85%, 4.08%, and 4.32%, respectively. Figure 1(A) shows a comparison of clinical outcomes between patients with and without anemia. Patients with anemia had a significantly increased risk of ischemic stroke/TIA, major bleeding, heart failure, and death compared to those without anemia. Figure 1(B) shows sensitivity analysis with anemia categorized into three groups: no anemia, mild anemia, and moderate to severe anemia. The results of that analysis were similar to those observed from the anemia versus no anemia analysis. The rate of clinical outcomes according to anemia status is shown in Supplemental Table S1.

### 3.2 Multivariate analysis and survival analysis

**FIGURE 2:**
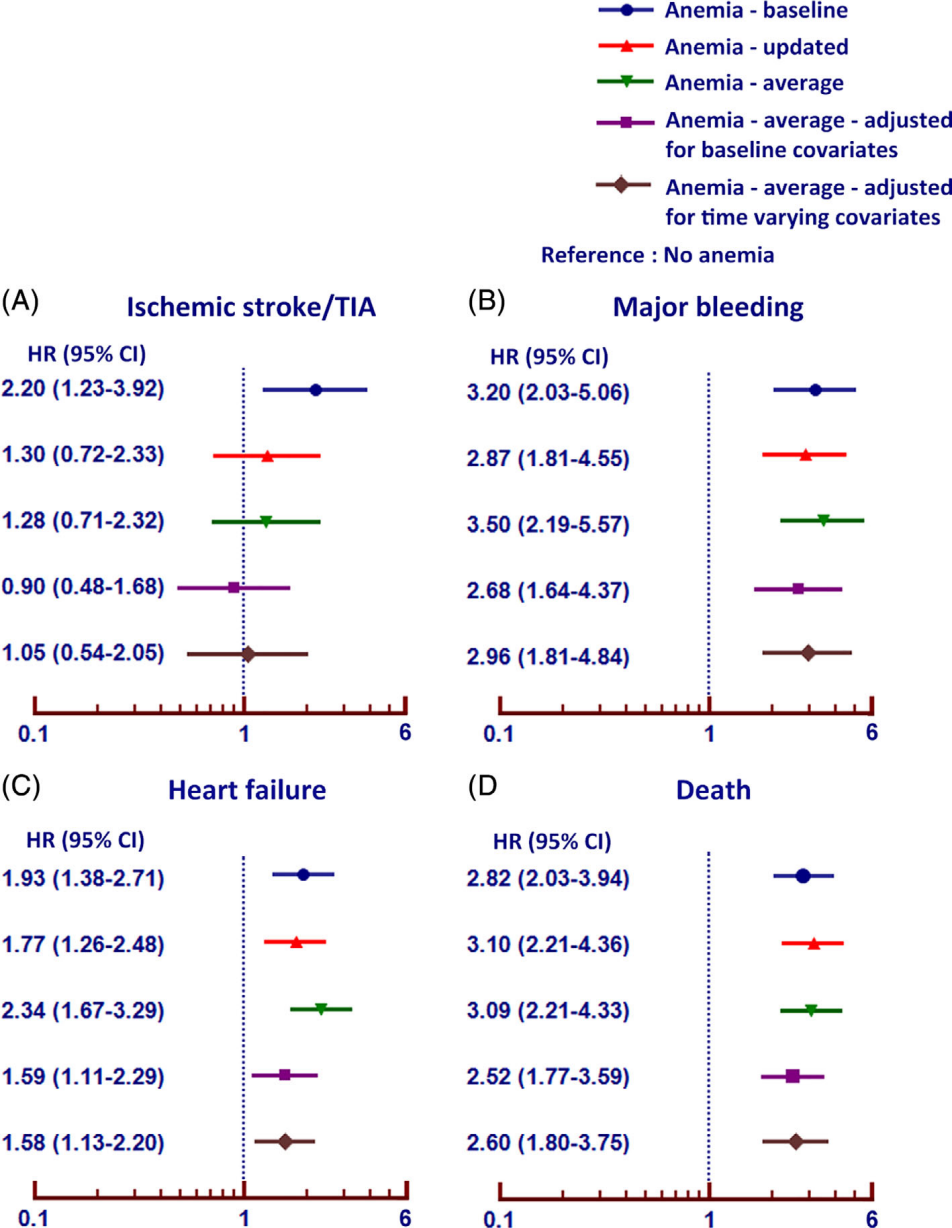
Unadjusted and adjusted hazard ratio and 95% confidence interval (CI) of anemia for increased risk of ischemic stroke/transient ischemic attack (TIA), major bleeding, heart failure, and death including the adjustment of time varying covariates

After adjusting for baseline and time varying covariates, anemia remained a significant predictor of major bleeding, heart failure, and death, but not for ischemic stroke/TIA (Forest plot shown in Figure 2). We also performed sensitivity analysis by using average hemoglobin from every visit to classify anemia. The results are demonstrated in Figure 2. Anemia remained independent predictor of clinical outcomes.

**FIGURE 3:**
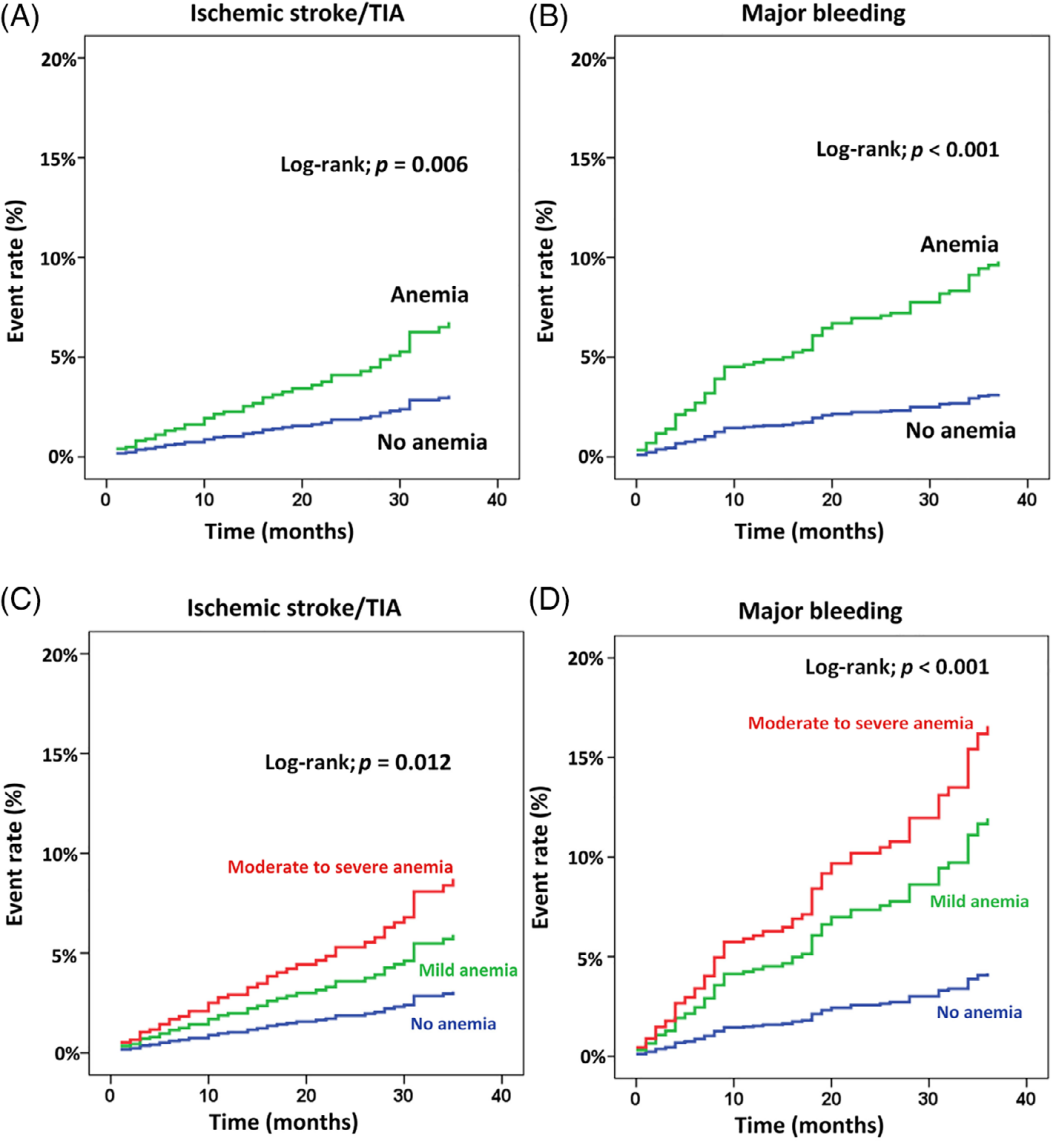
Cumulative event rate of (A) ischemic stroke/transient ischemic attack (TIA), and (B) major bleeding by anemia status. Cumulative event rate of (C) ischemic stroke/TIA, and (D) major bleeding compared among patients with moderate or severe anemia, mild anemia, and no anemia. A *p*‐value <.05 indicates statistical significance

Kaplan–Meier graph shows the difference in the rate of ischemia stroke/TIA and major bleeding between patients with anemia and patients without anemia during the follow‐up period (Figure 3(A)). The curves of the two groups become increasingly separated as the follow‐up time increases. Sensitivity analysis of anemia categorized into three groups was also performed. That analysis revealed a similar significant effect of anemia on clinical outcomes (Figure 3(B)). Effect of anemia on major bleeding, heart failure, and death persisted after adjusting for age, gender, comorbid conditions, and antithrombotic medications.

### 3.3 Anemia as a continuous variable

**FIGURE 4:**
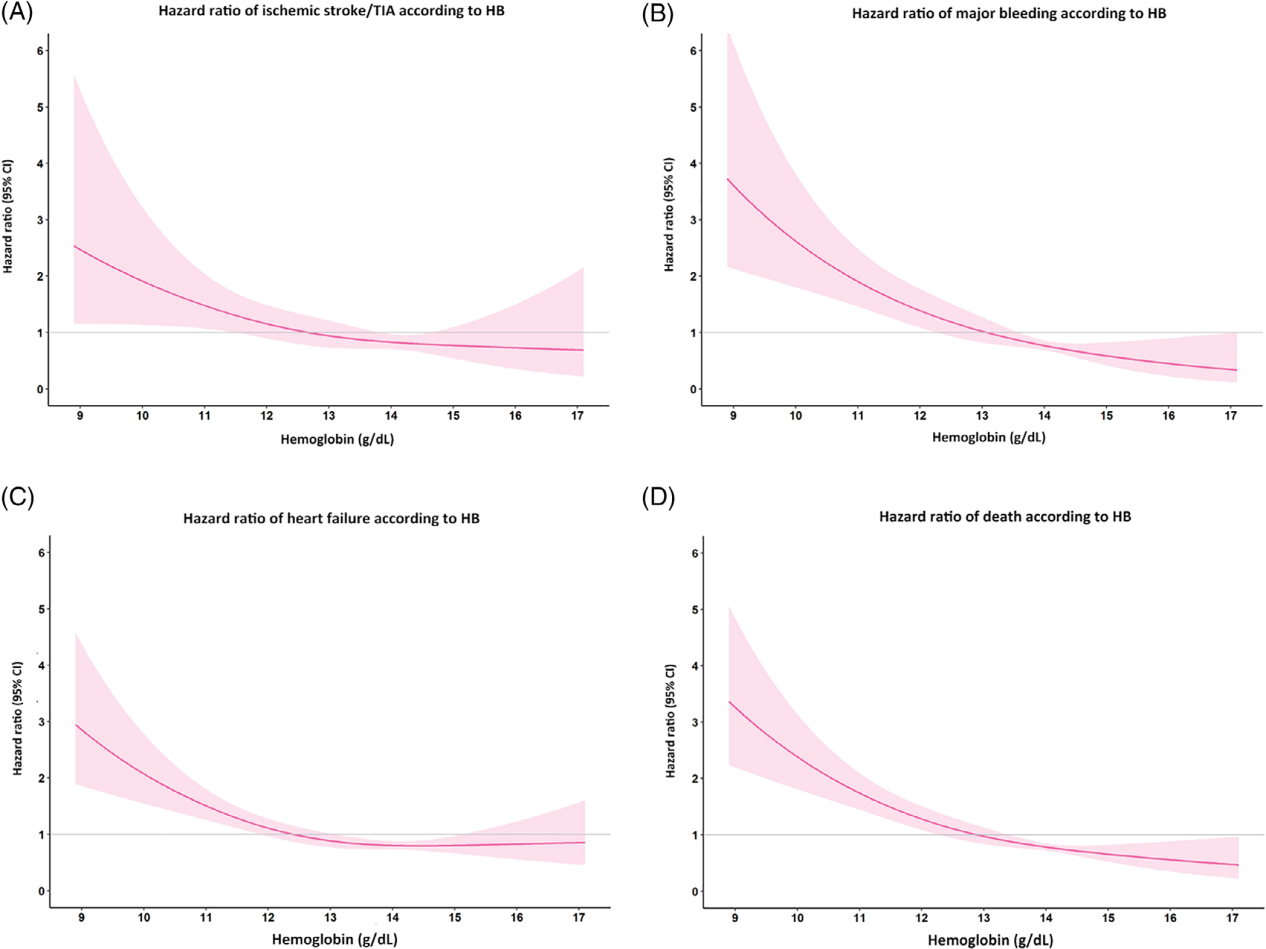
Restricted cubic spline graph showing hazard ratio and 95% confidence interval (CI) of hemoglobin (HB) as continuous data with (A) ischemic stroke/transient ischemic attack (TIA); (B) major bleeding; (C) heart failure, and, (D) death

When anemia was analyzed as continuous data by treating hemoglobin levels as continuous data, restricted cubic spline graphs showed that the risk of ischemic stroke/TIA, major bleeding, and death increased when hemoglobin level went below 13 g/dl, and markedly increased when hemoglobin levels were below 10 g/dl (Figure 4).

### 3.4 Effect of OAC

Supplemental Figure S1A shows the rate of ischemic stroke/TIA, and major bleeding in patients with and without anemia according to OAC status. Overall, OAC use was observed in 1169 patients (74.8%). Of those, 385 (32.9%) patients had anemia, and 784 (67.1%) patients did not have anemia (*p* = .741). Benefit was demonstrated only in patients without anemia. The risk of major bleeding significantly increased in patients with anemia, especially in those taking OAC. However, in our study population, warfarin was used in 88.9% of those who were on OAC. The average TTR was 52.1 ± 26.1. TTR was 49.0 ± 25.8% in patients with anemia compared to 53.7 ± 26.1% in those without anemia (*p* = .009). TTR ≥65% was observed in 28.4% of patients with anemia, and in 36.4% of patients without anemia (*p* = .012); 50.7 ± 25.4 in mild anemia and 45.4 ± 26.4 in moderate to severe anemia. Non‐vitamin K antagonist oral anticoagulants (NOACs) were used in 130 patients (11.1% of patients who were on OAC). The discontinuation rate was 89 (5.7%); 97(6.2%) for warfarin and 6(0.4%) for NOACs. OAC discontinuation was 38 (9.9%) in anemia and 51 (6.5%) in no anemia (*p* = .041), (24 [9.0%] in mild anemia and 14 [12.0%] in moderate to severe anemia). Among patients with anemia, the rate of ischemic stroke/TIA was 5.1% among those taking warfarin, and 0% among those taking NOACs (*p* = .176). The rate of major bleeding was 10.8% among warfarin users, and 5.9% among NOAC users (*p* = .367) in anemia group (Supplemental Figure S1B). Although NOACs seemed to reduce the rate of ischemic stroke/TIA and major bleeding compared to warfarin, no statistically significant difference between groups was observed due to the small sample size in the NOACs group. We should not make conclusion for NOAC data since that sample size was small in NOAC group.

## 4 DISCUSSION

The results of this nationwide NVAF registry in Thailand demonstrated that 33% of the study population had anemia. Anemia status increased the risk of ischemic stroke/TIA, major bleeding, heart failure, and death. After adjusting for potential confounders, anemia remained an independent factor that increased the risk of these adverse outcomes, except for ischemic stroke/TIA.

Anemia might be a risk factor for adverse events in many cardiovascular conditions, such as heart failure,^13^, ^14^ CAD after acute coronary syndrome^15^ or percutaneous coronary intervention (PCI), especially when the patient requires antiplatelet medication.^16^ It might also increase the risk of bleeding complication. Most patients with NVAF need OAC for stroke prevention.^2^, ^3^, ^4^ Anemia could affect physician or patient decision regarding whether or not to use OAC.^7^ It may also influence the choice of OAC, and when patients were on warfarin, anemia influenced the level of anticoagulation control leading to a poor TTR.^7^ The TTR among patients who were on warfarin in our study was also lower in patients with anemia compared to those without. This finding is consistent with that reported from a previous study.^7^

Data from 18 734 NVAF patients in a Danish registry revealed rates of mild and moderate to severe anemia of 20% and 14%, respectively.^7^ The prevalence of anemia in NVAF patients in that study was similar to the 33% prevalence of anemia we observed in the present study. Prevalence of anemia in Asian population was reported to be approximately 28–31%.^17^ The Danish study reported that moderate to severe anemia increased the risk of major bleeding, and that OAC had no benefit for stroke prevention among those with moderate to severe anemia. These findings are similar to the results from our study, except that data from our study demonstrated that mild anemia can increase the risk of major bleeding, heart failure, and death, and moderate to severe anemia further increases those risks. Restricted cubic spline graph demonstrated that the risk of these adverse outcomes increased when hemoglobin levels were below 13 g/dl, and increased even more dramatically when hemoglobin levels were below 10 g/dl. Results from the Danish registry showed that among anemic NVAF patients who were on warfarin, the TTR was 9% lower than among non‐anemic NVAF. The results from our study also showed a difference in TTR between patients with and without anemia, but the magnitude of difference between groups in our study was smaller (5% difference).

Post hoc analysis of data from the ARISTOTLE study, which included more than 18 000 patients with NVAF, showed a prevalence of anemia of 13%,^6^ which is significantly lower than results from our study. Anemia increased the risk of major bleeding and death in that study with hazard ratios (HRs) of 1.9 and 1.7, respectively, both of which are lower than the 3.2 and 2.8 HRs from our study. Anemia had no effect on the rate of ischemic stroke in the ARISTOTLE study (HR: 0.9), while our study showed anemia increased the risk of ischemic stroke/TIA with an HR of 2.2. That study found that apixaban reduced ischemic stroke and major bleeding compared to warfarin both in patients with and without anemia.^6^ We were not able to draw any conclusions relative to the comparative efficacy of NOAC compared to warfarin in our study due to the small size of the NOAC group. However, our results showed that NOAC reduced the risk of ischemic stroke and major bleeding by approximately half compared to warfarin, which suggests may even benefit patients with anemia. Data specific to the use of NOAC in 8356 NVAF patients with anemia were reported from Taiwan.^9^ That study found that NOAC could reduce the rate of major bleeding, but the rate of ischemic stroke was similar to that observed in patients taking warfarin among patients with anemia defined as hemoglobin level below 10 g/dl. Our study supported the results from Taiwan.

Anemia was reported to be a significant predictor of cardiac event in patients with NVAF even after adjusting for echocardiographic variables.^18^ Among 929 patients who were referred for PCI, 30% had anemia.^19^ Anemia was an independent risk factor for ischemic stroke, bleeding, and death.^19^

Compared to results of previous studies, Westenbrink et al^6^ demonstrated an increased risk of major bleeding and death but no effect on stroke risk. Our study showed that anemia increased risk of major bleeding and death. Anemia was not associated with an increased ischemic stroke risk after multivariate analysis. Bonde el al^7^ reported that anemia increased risk of major bleeding. However, the increased risk of ischemic stroke related to anemia was observed only in patients who received OAC. The possible explanation of increased risk of ischemic stroke from anemia might be related to the lower TTR in patients with anemia due to the fear of bleeding from physician. Besides, it has been reported that the rate of premature permanent discontinuation of warfarin was higher than apixaban from ARISTOTLE study with the discontinuation rate of warfarin was 23% at the median follow up of 7 months.^20^ Results of our study showed that OAC may not reduce risk of ischemic stroke in patients with anemia and TTR was lower in patients with anemia. Therefore, the implication from our results, may be to encourage physician to consider carefully for the benefit and risk of OAC before starting OAC therapy. When OAC was prescribed, physicians need to closely monitor INR target in patients with anemia. Alternative treatment would be to use NOAC instead of warfarin. Results of Bonde et al demonstrated that apixaban had benefit in the reduction of ischemic stroke and major bleeding in both patients with and without anemia. A recent study confirmed the benefit of NOAC in comparison to warfarin in the reduction of ischemic stroke and major bleeding.^21^ We also demonstrated an increased risk of heart failure in patients with NVAF and anemia. A previous study showed that elderly NVAF patients with anemia are less likely to receive OAC.^17^ However, data of our study demonstrated that the rate of OAC was not different between patients with or without anemia.

There are many mechanisms that link anemia with adverse outcome in patients with NVAF. Anemia may increase the risk of bleeding due to the underlying conditions of anemia, such as bone marrow failure and chronic kidney disease. Moreover, anemia may indicate a potential source of bleeding, such as gastrointestinal malignancy.^22^ The importance of anemia relative to the increased risk of bleeding in patients with NVAF is even more important in Asian population because Asians were found to be at increased risk for bleeding, especially when they were on OAC.^23^ Results from four NOAC trials also showed increased risk of bleeding among Asians.^24^ The rates of major bleeding and ICH in our study were 2.33% and 0.85% per year, which is higher than the rates reported in Western population from the GARFIELD registry^25^ and the Euro Heart Survey on Atrial Fibrillation.^26^ The increased risk of major bleeding in Asian population may be related to many factors, such as genetic predisposition,^27^ increased use of herbal medicine,^28^ and high rate of concomitant use of aspirin.^29^ Certain genetic factors in Asian population may explain warfarin dose variation.^27^ A previous study reported that when a patient develops bleeding while on OAC, the patient's physician should investigate for gastrointestinal malignancy that could be the source of bleeding.^30^

This study has some limitations. First, we used only anemia at the baseline visit as the criterion for including a patient in the anemia group. However, the laboratory result at the baseline visit may not reflect the hemoglobin level before clinical events. Second, the participating hospitals were mainly large community and university hospitals. Therefore, the results of this study may not be generalizable to other levels of care. However, the study sites are distributed all across Thailand. Third, warfarin was used in a majority of patients with OAC. Therefore, the results of our study may not be applied to population that receive NOAC in the majority. Fourth, we do not have the data on the etiology of anemia and cannot correlate the results of this study to patients with correctable and uncorrectable cause of anemia. However, we had data on history of major bleeding. Thirty‐seven patients (2.4%) had a history of major bleeding. History of major bleeding was presence in 22 (4.2%) of anemia group, and 15 (1.4%) in no anemia group (*p* = .001). Among patients with history of major bleeding, the bleeding sites were as follows: Intracranial 6 (16.2%), intra‐ocular 1 (2.7%), intra‐muscular 3 (8.1%), upper gastrointestinal 16 (43.2%), lower gastrointestinal 6 (16.2%), hematuria 4 (10.8%), and others 2 (5.4%). Lastly, we did not collect data on the impact on outcome of the correction of anemia during follow‐up.

The major strength of this study is its prospective registry design. More specifically, the study was designed before the enrollment of patients and data collection. Another important strength is that all primary outcome events were adjudicated by the study team before they were included for analysis.

## 5 FUTURE DIRECTIONS

More studies are needed to confirm the benefit of OAC especially NOAC in stroke prevention in patients with anemia. Future studies are needed to focus on the safely of OAC in patients with anemia. Net clinical benefit should be demonstrated for warfarin and NOAC in patients with anemia. For warfarin, more data are needed to look at the relation of TTR and risk of stroke and major bleeding. These future data should also include significant proportion of Asian population since Asian patients may behave differently from Western population.

## 6 CONCLUSION

Anemia was identified as an independent risk factor for major bleeding, heart failure, and death in patients with NVAF. OAC may not prevent ischemic stroke in patients with anemia, but it increases the risk of major bleeding. TTR was lower in patients with anemia compared to those without.

## CONFLICT OF INTEREST

The authors hereby declare no personal or professional conflicts of interest relating to any aspect of this study.

## AUTHOR CONTRIBUTIONS

Rungroj Krittayaphong: concept and design, data acquisition, interpretation of data, manuscript preparation, manuscript revision, and manuscript review; Satchana Pumprueg, Tomon Thongsri, Weerapan Wiwatworapan, Thaworn Choochunklin: data acquisition, manuscript revision, and manuscript review; Pontawee Kaewkumdee: data acquisition, interpretation of data, manuscript preparation, manuscript revision, and manuscript review and, Ahthit Yindeengam: data analysis, manuscript revision, and manuscript review. All authors read and approved the final manuscript, and approved the submission of this manuscript for journal publication.

## Supporting information


**Appendix** **S1**: Supporting information

## Data Availability

The dataset that was used to support the results and conclusion of this study are included within the manuscript. The additional data are available upon reasonable request.
