# Supplementary material for: Impact of anemia on clinical outcomes of patients with atrial fibrillation: The COOL‐AF registry
Source: Clin Cardiol. 2021 Feb 4;44(3):415–23. doi: 10.1002/clc.23559 (PMC7943899; doi:10.1002/clc.23559)

Supplementary materials

Supplemental Table S1. Rate of clinical outcomes according to anemia status

Anemia status	Number of Patients	Number of events	100 Person-Years	Rate per 100 Person-Years (95% CI)
**Ischemic stroke/TIA**				
Anemia	518	23	22.71	2.21 (1.40-3.32)
No anemia	1044	23	10.40	1.01 (0.64-1.52)
**Major bleeding**				
Anemia	518	46	22.71	4.42 (3.24-5.90)
No anemia	1044	31	10.40	1.37 (0.93-1.94)
**Heart failure**				
Anemia	518	63	22.71	6.06 (4.66-7.75)
No anemia	1044	72	10.40	3.17 (2.48-3.99)
**Death**				
Anemia	518	81	22.71	7.79 (6.19-9.68)
No anemia	1044	62	10.40	2.73 (2.09-3.50)

CI = confidence interval, TIA = transient schemic attack

Supplemental Figure S1. Effect of oral anticoagulant (OAC) on clinical outcomes. Rate of ischemic stroke (IS)/transient ischemic attack (TIA), major bleeding (MB), and intracerebral hemorrhage (ICH) according to A. anemia status and OAC treatment B. anemia status and warfarin or NOAC.

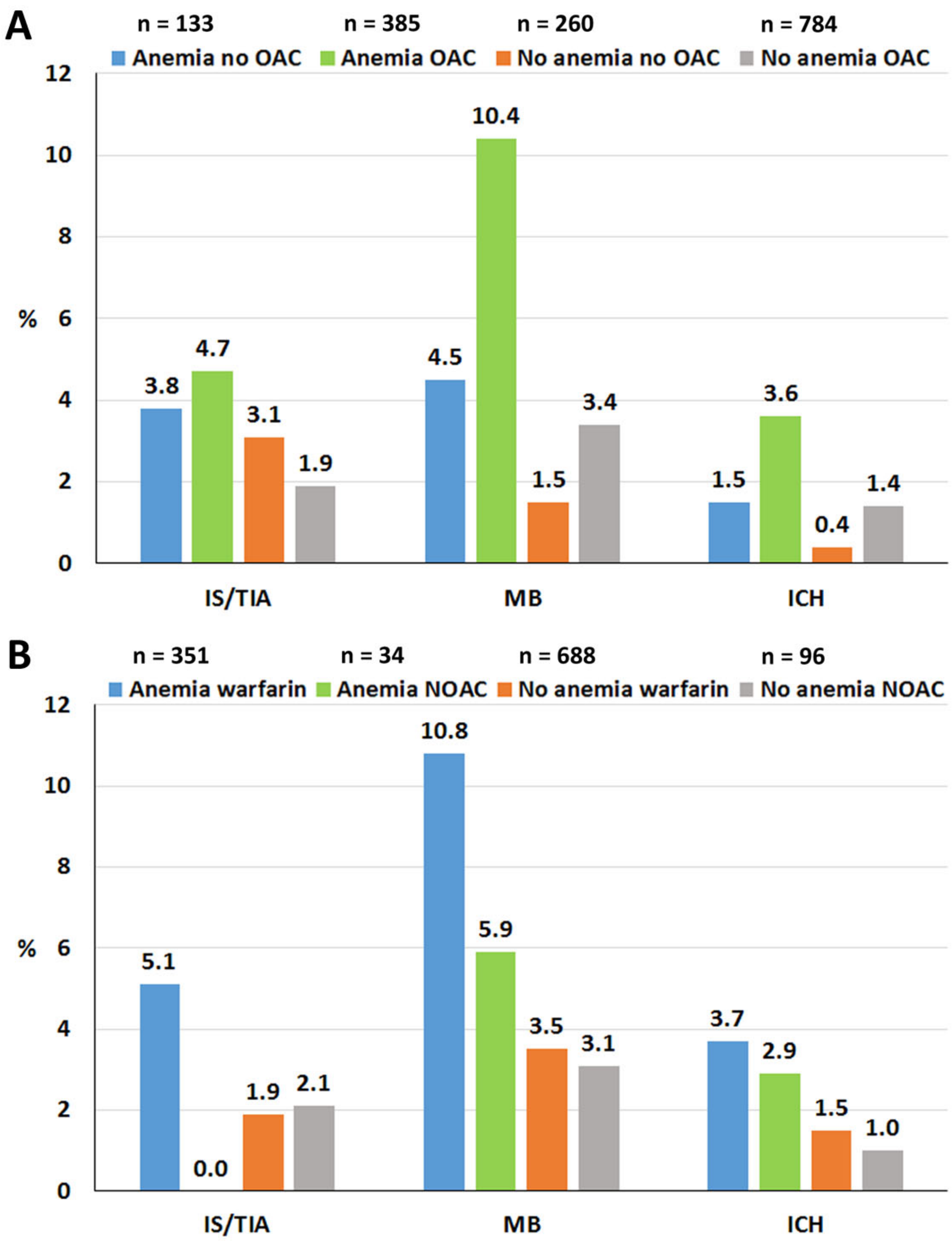

Supplement: Supplementary file 1 — Appendix S1: Supporting information [file CLC-44-415-s001.pdf]
